# Biomechanical, Physiological and Anthropometric Determinants of Backstroke Swimming Performance: A Systematic Review

**DOI:** 10.1186/s40798-025-00868-z

**Published:** 2025-06-08

**Authors:** José M. González-Ravé, Fernando González-Mohino, Francisco Hermosilla Perona, Victor Rodrigo-Carranza, Inmaculada Yustres, David B. Pyne

**Affiliations:** 1 Sport Training Laboratory, Faculty of Sports Sciences, University of Castilla-La Mancha, Carlos III Avenue, 45008 Toledo, Spain; 2 Department of Physical Activity and Sports Science, Alfonso X El Sabio University, 28691 Madrid, Spain; 3 Footwear Innovation Production, Tempe-Inditex, 03203 Alicante, Spain; 4 Universidad Francisco de Vitoria, Madrid, Spain; 5 Research Institute for Sport and Exercise, University of Canberra, Canberra, Australia

**Keywords:** Swimmers, Training, Backstroke, Performance

## Abstract

**Background:**

Backstroke swimming is one of the four competitive strokes contested at international swimming events, and the second-slowest stroke after breaststroke. Achieving success in competition depends on the intricate interplay of various factors, and for top-ranked athletes, subtle differences can be decisive in determining the race outcome. The aim of this study is to identify the main energetic, biomechanical, and physiological factors influencing elite backstroke swimming performance in 50, 100, and 200-m events.

**Methods:**

The Preferred Reporting Items for Systematic Reviews and Meta-Analyses (PRISMA) guidelines were used to identify relevant studies. A literature search on 3 databases (PubMed, Scopus, Web of Science) was finalised on September 26, 2024.

**Results:**

A total of 938 studies were identified, and finally 35 studies met the inclusion criteria. The swimmers (n = 507 participants, n = 188 women) were classified as Tier 3 (Highly Trained/National Level) or Tier 4 (International Level). Studies included exhibited a low risk of bias following the Hindle scale (11 ± 2 points). All the studies were observational. Reference values have been provided with normative blood lactate, kinematics, race pace, performance testing and anthropometric characteristics for comparison purposes in 50, 100, and 200-m events. Post-race blood lactate concentrations were lower in the 50 m compared to the 100 m and 200 m events. Differences were evident in stroke rate (SR) reference ranges between events (50 m, 100 m, 200 m), anthropometric profiles (swimmers’ height and hand, foot, and leg length), and pacing profiles (50 m: all-out trend; 100 m: positive profile; 200 m: parabolic approach or fast-track strategy).

**Conclusions:**

There is a distinctive physiological and biomechanical pattern for distances from 50-m to 200-m in backstroke swimming. The data provide athletes and coaches with normative reference values for blood lactate, kinematics, race pace, and anthropometric measures.

## Key Points

- A distinctive physiological and biomechanical pattern for distances has been described from 50 to 200-m in backstroke swimming
- Post-race blood lactate values typically range from 9.1–11.1, 12.4–13.9, and 13.0–14.0 mmol L^−1^ for 50, 100, and 200-m/yard backstroke events. Pacing profiles, physiological and stroke kinematics measures differ between the 50, 100, and 200-m/yard backstroke events.
- Coaches should focus on training exercises that increase stroke length and decrease energy cost, especially in the 200-m backstroke.
- Coaches and practitioners should consider individual variations in anthropometric characteristics when identifying backstroke swimmers (body height, hand and foot length in the 50-m, and sitting height, leg length, and forearm and arm girths for the 100–200-m events).

## Background

Backstroke swimming is one of the four competitive strokes contested at international swimming events, and the second-slowest stroke after breaststroke. The program of backstroke events includes races over 100-m and 200-m in the Olympics, with an additional 50-m event in World Championships [1] for both men and women. The 2024 world rankings provide the best times for men in the 50-m (23.96 s: 949 AQUA points), 100-m (52.00 s: 964 AQUA points), and 200-m (1:54.51: 933 AQUA points) events. For women, the best times are in the 50-m (26.86: 1000 AQUA points), 100-m (57.13: 1010 AQUA points), and 200-m (2:03.30: 996 AQUA points) events. These exceptional performances result from a complex process of training, which includes the detailed analysis of all variables involved in backstroke swimming for achieving medal-winning performances.

The variation in event distances between 50- and 200-m yields differences in energy system requirements, despite athletes performing the same stroke [2]. In the past, the performance enhancement was underpinned by improving mostly the swim stroke (i.e. clean swimming) [3, 4], while nowadays the focus is shifting more towards other phases of the race such as start, turn and finish [5]. The world records in men’s and women’s backstroke events have improved by approximately 4% over the last two decades (since the year 2000), and further year-on-year improvements will come in part from ongoing improvements in biomechanical, physiological and anthropometric determinants of backstroke swimming performance. While the majority of these improvements will relate to the expertise and experience of coaches, there is an opportunity to supplement this knowledge with the evidence contained from experimental (research) studies.

Backstroke is characterized by a cyclic movement subdivided into two phases: an aerial phase and an underwater phase. The first phase is non-propulsive, while the second phase is further subdivided into propulsive and non-propulsive subphases [6]. The kinematic characteristics of backstroke include alternating limb motions and body roll around the longitudinal axis [7]. Swimming velocity is determined by stroke rate (SR), which is the number of stroke cycles per minute, and stroke length (SL), representing the distance covered with each stroke cycle. The length of a stroke is influenced by technical skills and generally considered an indicator of motor effectiveness [8, 9], whereas SR is more closely associated with neuromotor and energetic capabilities [9]. Relationships between energy costs of swimming, distance per stroke, SR and the velocity of swimming can be modified in an individual swimmer with training [10]. Barbosa et al. [11] affirmed that backstroke was the second most efficient competitive style after freestyle, at all selected velocities. This relative efficiency is related to lower intracyclic velocity variation in backstroke compared with the other competitive strokes. SR increases are associated with increases in energy cost, even when controlling for velocity, while an increase in SL is associated with a decrease in energy cost in the 200-m backstroke [12]. These biomechanical patterns are also influenced by anthropometric factors [13, 14]. For instance, having greater height and arm span—along with high body mass and lean body mass—positively contributes to better SL and stroke index values.

To identify areas for improvement, swimmers and coaches need clear and easily understandable information on swimming performances, particularly in less-studied strokes such as backstroke. For example, of the 26 swimmers recruited by Barbosa et al. [11], only five were backstroke specialists. By identifying areas for improvement, swimmers can target specific aspects of their training to enhance their skills and achieve better results from a biomechanical or physiological perspective. The final race time is influenced to a greater or lesser extent by the start, turn and finish depending on the race distance, with the influence greater the shorter the distance of the event [15]. In 100-m backstroke, times spent in the turning and starting phases of swimming races are strongly related to swimming performance (including underwater gliding and underwater leg movements) [16]. In short events (e.g. 100-m events), the start and turn account for nearly a third of the final race time [5, 17]. In terms of pacing patterns in the 200-m backstroke, swimmers typically display a fast-start even profile, exhibiting a substantially faster first section as well as a faster second split time compared to lap splits 3 and 4 [18]. This ability to adjust SL and frequency seems to be learned as part of training for competition over a season [19, 20]. In terms of kinematic measurements, one study reported the velocities in 200-m backstrokes ranged from 1.10 to 1.39 m s^−1^, being 70% of aerobic and 30% of anaerobic energy systems [12]. While these studies provide useful technical information the continuing evolution of training and competitive backstroke necessitates a comprehensive and systematic evaluation of the relevant studies for the benefits of athletes, coaches, sports science practitioners, and researchers.

Achieving success in competition depends on the interplay of various factors, and for top-ranked athletes, subtle differences can be decisive in determining the competition outcome. Despite the technical constraints placed on athletes during different swimming events such as breaststroke [21], a level of variability based on temporal characteristics, coordination patterns, neuromuscular activity, and pacing profiles is still possible between individuals. A review of the current literature on these aspects will help the swimming community understand what features have been studied, and what variables need to be improved in training and competition. As no previous review of backstroke swimming has been conducted, a comprehensive analysis of backstroke studies will inform future training practices and research priorities. The objective of this study was to systematically review anthropometric, energetic, biomechanical, and physiological factors influencing elite backstroke swimming performance in 50, 100 and 200-m events.

## Methods

### Search Strategy

The systematic review was conducted following the guidelines provided in the Preferred Reporting Items for Systematic Reviews and Meta-Analyses (PRISMA) statement [22]. A comprehensive search of three online databases (PubMed, Scopus, Web of Science) was completed on February 12, 2025, by two independent researchers (FH and JMGR). Title, abstract, and keyword fields were screened using the following search combinations: “Backstroke AND swimming”, “Backstroke AND Stroke rate”, “Backstroke AND Stroke length”, “Backstroke AND velocity OR speed”, “Backstroke AND bioenergetics”, “Backstroke AND lactate”, “Backstroke AND energetics”, “Backstroke AND oxygen”, “Backstroke AND pacing”, Backstroke AND anthropometry”.

Searches were limited to human participants and articles written in English language. Two researchers (FH and JMGR) performed independently the identification, screening, eligibility, and inclusion of studies, with disagreement settled by a third researcher (IY). Where abstracts indicated that papers were potentially suitable, the full-text versions were obtained and included in the review if they fulfilled the selection criteria. Additional records were identified through other sources (such as manual searches through article reference lists) to ensure the inclusion of all the available published evidence.

### Inclusion and Exclusion Criteria

Studies were included in the systematic review based on the following criteria: (1) articles published in English language, and (2) experimental or case studies. The exclusion criteria were as follows: (1) non-research articles (editorials, commentaries, preprints, abstracts, proceedings, book reviews, etc.), (2) articles not focused on backstroke, (3) articles that did not provide details about biomechanics or physiological aspects, (4) participants under 13 years old or masters swimmers, and (5) articles where data were not in line with the research question.

### Data Extraction

Two independent researchers (FH and JMGR) independently extracted characteristics of training protocols and results using a standardized form, with disagreement settled by a third researcher (IY). Data extracted from all the eligible studies were classified into different groups regarding the data presented in the study. The groups were organized according to the following terms: “Stroke rate”, “Stroke length”, “Swimming velocity”, “Stroke coordination”, “Lactate”, “VO_2max_ or economy”, “Pacing”, “Underwater undulatory swimming”, “Critical velocity” and “Anthropometrics”.

### Assessment of Methodological Quality and Risk of Bias

Two independent reviewers (FH and JMGR) analysed the quality of included studies. The quality of eligible studies was assessed using the risk of bias scale developed by Hindle and colleagues [23]. This scale is based on other evaluation checklists and has been used previously for assessment of sports research [24, 25]. Sixteen standards were used to evaluate article quality: three standards on study design, four standards on sample characteristics, four standards on methodology, and five standards on results and discussion. A detailed outline of assessment criteria is provided in Table 1. One point was awarded for each standard met to a maximum total of 16 points. No half points were awarded. Risk of bias score was subsequently determined using the total number of points awarded. Articles scoring 11–16 points were categorised as a low bias risk. Articles scoring 6–10 points were categorised as satisfactory bias risk, while a study with a score 0–5 was categorised as a high risk of bias.

**Table 1:** Physiological and anthropometry results of studies examining backstroke swimming

Study	*n* (M/F)	Age (years)	Level sample			
				Intervention	Measures	Results
Carvalho et al. [26]#	72 (47/25)	20.3 ± 3.1 18.3 ± 2.5	Tier 3. 811 ± 32 and 808 ± 39 FINA points of best competitive performance	5 × 200 m intermittent incremental protocol, with 0.05 m/s increase per step/30 s rest	Lactate, stroke rate, stroke length, swim velocity	Anaerobic threshold 3.9 ± 1.1 mmol/L corresponding with stroke rate and stroke length inflection points
Neiva et al. [33]#	8 (8/0)	20.2 ± 3.2	Tier 3. National swimmers with a training experience of 11.13 ± 3.62 years, training from 6 to 10 times per week	Swimmers performed maximal 15, 25, 37.5 and 50 m in the 4 swimming techniques to determine critical velocity from the distance-time relationship	Swim velocity, critical velocity	Anaerobic critical velocity was 1.53 ± 0.05 m/s for backstroke
Ozkadi et al. [27]#	40 (20/20)	16.5 ± 0.5	Tier 2. Swimmers had at least three years of experience	Anthropometric and body composition, and competition times	Anthropometrics, stroke rate, stroke length, swim velocity	Standing horizontal jump, and aerobic endurance, sit and reach flexibility, agility, and balance were significantly prominent predictive variables
Klentrou and Montpetit [28]	38 (22/16)	14.7 ± 1.4 16.5 ± 2.5	Tier 2. Trained swimmers with a VO_2max_ of 52.2 ± 5.9 and 59.3 ± 5.2 for women and men	Energy cost of swimming was measure at different velocities (1.0, 1.1, and 1.2 m/s) in men and women	Swim velocity, VO_2_	Mean VO_2_ of males ↑compared with females, but identical slopes of the regression. Increased velocity in both groups related to ↑in force and a ↓ in distance. Costs per stroke in males were ↑ compared with females
Capelliet al [34]#	20 (20/0)	18.9 ± 0.9	Tier 3. Elite male college swimmers with a VO_2max_ of 4.29 ± 0.49 L min	Energy cost per unit of distance was assessed at sub-maximal velocities and at critical velocity	Swim velocity, lactate, VO_2_	Energy cost per unit of distance and lactate was a continuous function of the velocity increasing exponentially
Holmér [35]#	2 (2/0)	20.0 ± 0.0	Tier 3. Male elite swimmers	Maximal swimming was preceded by 2 min of swimming at a submaximal velocity of 70 to 80% of the swimmer's maximal velocity	Swim velocity, VO_2_, heart rate and lactate	Highest values for VO_2_, heart rate and blood lactate when swimming the whole stroke, and lowest with arm strokes only. At higher velocities body drag was 0.5 to 0.9 kp lower when arms or legs were supported by a cork plate
Barbosa et al. [11]#	26 (18/8)	–	Tier 4. International swimmers	Incremental test of 200 m to exhaustion	Swim velocity, VO_2_	Energy cost was ↑ in breaststroke compared with backstroke at 1.2, 1.4 and 1.6 m/s but not at 1.0 m/s
Rejman et al. [29]#	130 (95/35)	19.5 ± 2.9 18.4 ± 2.8	Tier 4. Polish, Norwegian, and Portuguese national swimming	A set of anthropometric variables was used to predict swimming velocity performance through a regression model	Swim velocity, anthropometrics	A large trunk “v-shape” morphology and large hand areas important factors in determining velocity
Sammoud et al. [30]	63 (30/33)	13.9 ± 0.6 13.0 ± 1.2	Tier 2. Young swimmers performing 5–6 training sessions per week (distance 4000 ± 1000-m per session; 8 ± 1 h per week)	A multiplicative allometric model of 100 m backstroke mean velocity performance and the different somatic measurements	Swim velocity, anthropometrics	Stature and body mass did not contribute to the performance model. Advantage of longer levers was limb-specific rather than whole-body
Makar and Bielec [32]	1 (0/1)	16.0 ± 0.0	Tier 3.A female member of the Polish National Junior Swimming Team	Ten trials of a 5 × 200 m backstroke step test	Swim velocity, lactate and glucose	During maximal effort, lactate increased 5.1–13.1 mmol/l, glucose concentration 6.6–8.1 mmol/l. Shape of lactate and glucose curves improved
Smith et al. [36]	16 (16/0)	> 16	Tier 3. Senior National level male competitive swimmers	Energy cost of swimming, velocity and stroke rate were measured during backstroke swimming at velocities from 1.0 to 1.4 m/s	Stroke rate, swim velocity, VO_2_	VO_2_ related to body mass, height, arm span and speed. ↑ distances per stroke associated with ↓ O_2_ cost, and ↑maximal performances. Submaximal VO_2_ related to 100 m and 200 m performance
di Prampero [37]#	20 (20/0)	18.9 ± 0.9	Tier 4. Elite male swimmers (VO_2max_ of 4.12 ± 0.75 L min)	Critical velocity was compared with the velocity maintained on the basis of the subject’s VO_2_max	VO_2_, critical velocity	Critical velocity 16% ↓ than velocity maintained on the basis of VO_2_max, and the distance covered at the expense of the anaerobic capacity (11.6 m) was ↓ than anaerobic distance
Barbosa et al. [12]#	5 (5/0)	19.0 ± 1.4	Tier 3. Portuguese national team swimmers	An intermittent set of n × 200 m swims (n < 8) with increasing velocity	VO_2_, Lactate, Stroke rate, stroke length, swim velocity	Relationship between stroke frequency and energy cost (R^2^ = 0.14, P = 0.05), and correlation between speed and stroke frequency controlling for stroke length (R = 0.64, P < 0.01)
Vescovi et al. [31]#		20.2 ± 3.3	Tier 3. Swimmers of Canadian National Swimming Championships	Post race lactate measurement after the 50, 100 and 200 m final races (top 8)	Lactate	Post-race lactate values in 100–200 m events were ~ 13–14 mmol L and lower after 50 m races (~ 9 mmol L), with age effect on post-race lactate

m = metres, m/s = metres per second, VO_2_max = maximal oxygen uptake, L.min^−1^ = litres per minute, mmol.L = milimols per litre, O_2_ = oxygen, R^2^ = variance explained, ↓ = decreased, ↑ = increased, FINA = Federation Internationale de Natation. Swimmer classification: Tier 0 Sedentary, Tier 1 Recreationally Active, Tier 2 Trained/Developmental; Tier 3 Highly Trained/National Level; Tier 4 Elite/International Level; Tier 5 World Class according to the framework of McKay et al. [38]

#Articles including more than one stroke technique

## Results

### Characteristics of the Studies Selected

**Fig. 1:**
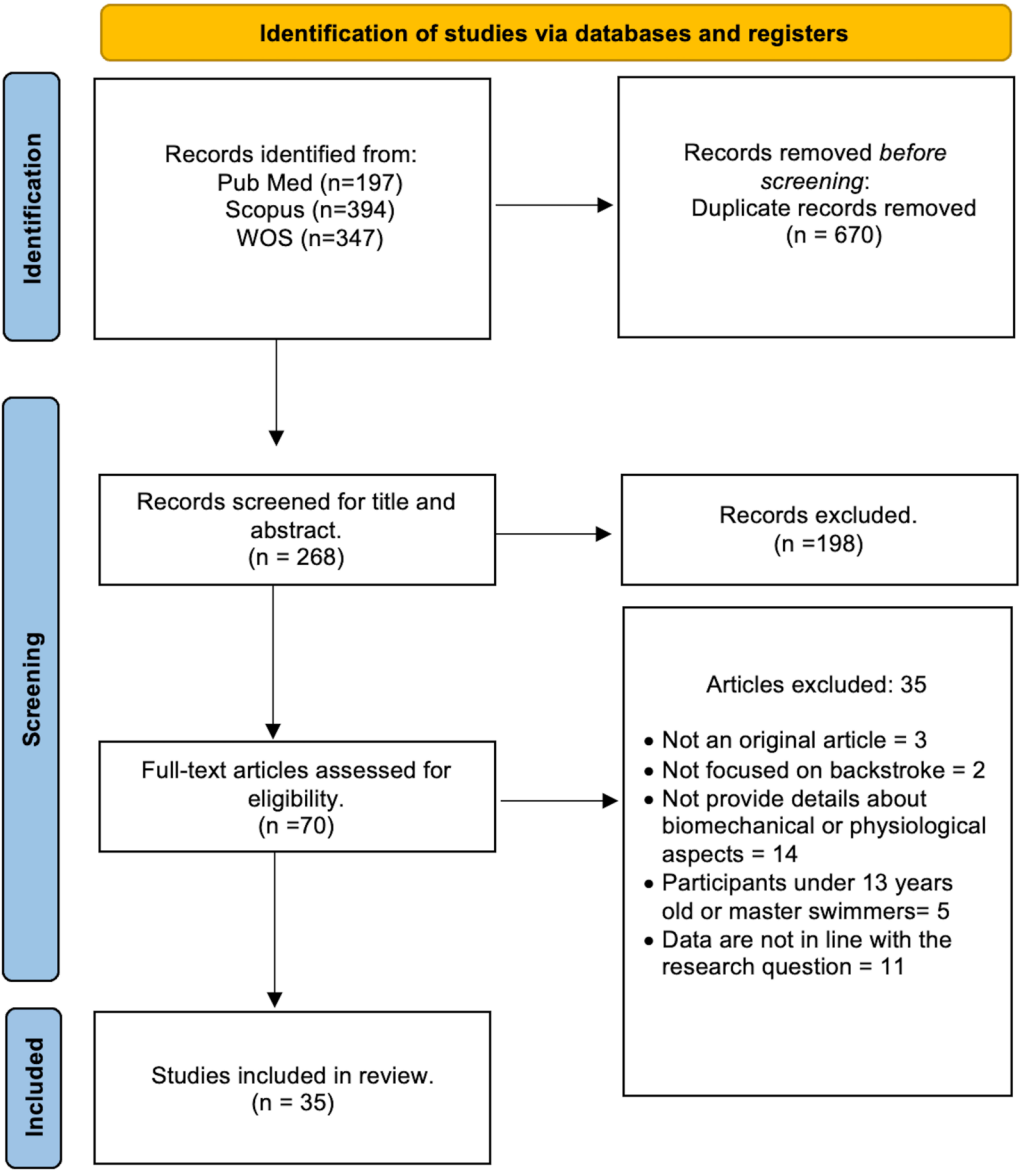
PRISMA flow diagram of the process used in selection of the journal articles included in the systematic review

A total of 938 studies were identified (Fig. 1). The reference list of selected manuscripts was also examined for other potentially eligible manuscripts. After the removal of duplicates and elimination of papers based on title and abstract screening, 268 manuscripts remained, 70 were assessed full text for eligibility, and a final total of 35 studies were included in the systematic review. The studies that did not match the eligibility criteria based on full-text screening were discarded for one or more of the following reasons: (1) studies not focused on backstroke, (2) studies that did not provide details about biomechanics or physiological aspects, (3) participants under 13 years old or master swimmers, (4) studies in which data are not in line with the research question, and (5) not an original article.

### Study Design

In this systematic review, we identified various types of study designs that contributed to the synthesis of evidence.

Most of the included studies, depending on the type of intervention, were observational (n = 27), followed by quasi-experimental (n = 8). Regarding the study time orientation, most included studies were cross-sectional (n = 33), and only two were longitudinal. Finally, based on the study purpose, fourteen were catalogued as comparative, nine as descriptive, and eight as correlational studies.

A total of 35 studies met all the inclusion requirements. None of the articles were cross-sectional studies. The included studies described various components of backstroke swimming: biomechanical parameters including SR (n = 19) [6, 12, 16, 17, 26, 36, 41, 45–52, 55, 56, 58, 60], SL (n = 18) [6, 12, 16, 17, 26, 41, 45–52, 55, 56, 58, 60], swim velocity (n = 28) [6, 11, 12, 16, 17, 26, 28–30, 32–36, 41, 45–53, 55, 56, 58, 60], pacing (n = 3) [18, 51, 56], coordination (n = 7) [6, 45, 46, 49, 54, 55, 58], underwater undulatory swimming (n = 2) [59, 60], and physiological parameters such as lactate (n = 4) [26, 31, 32, 34], oxygen/energy cost (n = 7) [11, 28, 34–37], critical speed (n = 4) [26, 33, 37, 41], and anthropometry (n = 3) [27, 29, 30]. 9 studies only analyzed specifically the backstroke stroke, while 26 studied different variables in more than one stroke (Tables 1 and 2).

**Table 2:** Kinematics and velocity results of studies of backstroke swimming

Study	*n* (M/F)		Age (years)	Level sample			
					Intervention	Measures	Results
Kolmogorov & Duplishcheva [53]#	20 (10/10)		20.9 ± 1.9 18.2 ± 2.1	Tier 4. Members of the Soviet national team training for the Goodwill Games	Passive and active drag at 1.45–1.72 m/s with a dynamometrical system	Swim velocity	Females active drag ranged from 66 to 38 N and men from 146 to 46 N
Lerda & Cardelli, [58] (2003)	36 (36/0)		22.7 ± 1.5	Tier 2. Expert and active backstroke swimmers	3 × 25 m near 100 m velocity with a passive rest period of 5 min measuring stroke kinematic	Stroke rate, stroke length, swim velocity, coordination	Swim velocity ↑ with duration of entry and catch and pull phases, but negatively correlated with the duration of clearing. Entry and catch phrase explaining 71% of the variance of swimming speed
Chollet et al. [49]#	14 (14/0)		19.0 ± 4.6	Tier 3. International-standard swimmers who performed to within 90% of the 100 m backstroke world record	4 × 25 m at the velocities corresponding to the 400 m, 200 m, 100 m, and 50 m events measuring stroke kinematics including the index of coordination	Stroke rate, stroke length, swim velocity, coordination	↑ in velocity = ↑ stroke rate, relative duration of arm pull, and index of coordination, and ↓ distance per stroke. Arm coordination was always in catch-up (index of coordination 12.9%)
Lerda et al. [55]	36 (18/18)		21.4 ± 1.5 20.6 ± 1.3	Tier 2. Students in Sport Sciences (not competitive swimmers), trained to swim twice a week for 20 weeks	3 × 25 m near 100 m velocity (± 2.5%) with complete rest. Stroking parameters and coordination indexes were measured. Index 1 = continuity between propulsive phases of each arm. Index 2 = the simultaneity between the beginning of the pull of one arm and of the recovery of the other arm	Stroke rate, stroke length, swim velocity, coordination index	Index 1 = women ↑ continuity than men. Performance explained by the increase in the entry and catch phases at the expense of clearing. For men, Index 2 = simultaneously is ↑ in faster than slower swimmers and correlated with performance. For women, the performance was explained by Index 1
Gonjo et al. [45]#	3 (3/0)		17.5 ± 1.00	Tier 2. Competitive swimmers	4 × 50 m trials at different velocities (83, 88, 93, and 100% of their maximum swimming velocity)	Stroke rate, stroke length, swim velocity, coordination,	Swimmers ↓ their whole-body roll and shoulder roll amplitude with an ↑ speed
Chollet et al. [48]#	203 (110/93)		–	Tier 3. Swimmers of French and Mediterranean championships	100 and 200 m competitions in a 50 m swimming pool	Stroke rate, stroke length, swim velocity, coordination	↓ velocity, stroke rate and stroke length in 200 m compared to 100 m in both sexes
Gonjo et al. [41]#	6 (6/0)		17.5 ± 1.0	Tier 3. National top 200 of 50, 100 and 200 m	Energy cost and kinematics using three-dimensional video to determine stroke parameters	Stroke rate, stroke length, swim velocity, critical velocity	No differences in stroke frequency/length and intra-cycle velocity fluctuation between techniques. ↓ 3D wrist and ankle velocities underwater and ↓ ankle vertical range of motion in front crawl than in backstroke. Hiigher propulsive efficiency in front crawl
Gonjo et al. [60]#	8 (8/0)		24.2 ± 3.6	Tier 4. Finalists in 2021 European long-course swimming championships	Eight digital video cameras recorded the races (200 m), and the video footage was manually analyzed	Stroke rate, stroke length, swim velocity	Breaststroke ↑ clean swimming velocity and ↑ distance per stroke than individual medley swimmers for both sexes. For backstroke and front crawl, specialists exhibited faster underwater velocities than individual medley swimmers
Veiga et al. [57]#	–		–	Tier 4. International Swimmers	The 100 and 200 m events during the FINA 2013 World Swimming Championships were filmed	Swim velocity	100 m race times were largely related to ↑ start velocities in men’s and 200 m in women’s. Changes in turn distances (especially in the last turn) improved performance in 200 m events
Cortesi et al. [6]	18 (10/8)		17.7 ± 2.8 18.3 ± 5.7	Tier 3. Trained 50 and 200 m backstrokes specialized in their preferred distance event for a minimum of 2 years	Two 25-m backstroke trials at 70% and 100% of maximum velocity	Stroke rate, stroke length, swim velocity, coordination	In the 50 m, the duration of the propulsive phase ↑ with ↑ swimming velocity. Both the pull and push phases fundamental in ↑ of duration of propulsive phase. 200 m specialists and 50 m distance swimmers can modify their arm stroke phases duration when ↑ swimming velocity in backstroke
Gonjo et al. [50]#	10 (10/0)		17.5 ± 1.0	Tier 3. Regularly trained (FINA point scoring was 600.20 ± 50.81)	Swimmers performed 50 m swims at four swimming velocities (83, 88, 93, and 100% of their backstroke maximum effort) and their whole-body motion was quantified by a 3D direct linear transformation algorithm	Stroke rate, stroke length, swim velocity,	Swimmers had 8.3% ↑ stroke length, 5.4% ↓ stroke frequency, 14.3%↓ the index of coordination, and 30.8% ↑froude efficiency in front crawl than backstroke = front crawl more efficient than backstroke. Backstroke had 25% ↑ active drag at 1.2 m/s than front crawl
Bartolomeu et al. [54]#	15 (15/0)		16.0 ± 2.9	Tier 2. Swimmers with training volumes of approximately 16,000 m per week, from regional to national level	4 × all-out bouts of 25 m (four strokes)	Coordination	All strokes and conditions presented contralateral limb asymmetries (peak force and mean force). Backstroke was the most asymmetric stroke
Hellard et al. [56]#	64 (0/64)		22.0 ± 3.0	Tier 4. Elite female swimmers (200 m events) of global championships	Differences between the first and second 100 m were calculated in the 200 m official event	Stroke rate, stroke length, swim velocity	Stroke rate and stroke length ↑, and velocity ↓ in the Olympic Group than the National group. Stroke rate variability depended upon interaction between biomechanical requisites and standard of practice
Oliveira et al. [52]#	78 (0/78)		–	Tier 4. Female swimmers competing in the four 50 m events of the 2021 European Championships	Velocity and stroke kinematics were analyzed in 5 pool section (0–15 m, 15–25 m, 25–35 m, 35–45 m and 45–50 m)	Stroke rate, stroke length, swim velocity	Swimming velocity exhibited a marked tier effect in all race sections and swimming strokes. Stroke frequency presented an overall tier effect
Morais et al. [17]#	78 (78/0)		–	Tier 4. Male swimmers who competed in the 50 m events (i.e., heats, semi-finals, and final) at the 2021 European Championships	Swimmers were split in two groups (better and poorer performances) and velocity and stroke kinematic were analyzed in 5 pool section (0–15 m, 15–25 m, 25–35 m, 35–45 m and 45–50 m)	Stroke rate, stroke length, swim velocity	Swimming velocity was the variable with ↑ variance in both groups. Velocity –time curve fitting for both groups suggested a cubic relationship. No difference in stroke frequency but stroke length higher at the beginning (15–25 m) of the event
Skorski et al. [18]#	34 (34/0)		22.8 ± 2.9	Tier 4. World’s top 50 rankings of the year 2010	Average race velocity (m/s) was calculated of 200 m heats and final events and in each split (50, 100, 150, 200 m) expressed relative to overall race velocity	Pacing	Percentage of the subject’s mean score were low in the first (CV 0.9–1.7%) and last Sects. (1.9–2.2%). Within-subject CV for changes between laps were between 0.9% and 2.6% in all finals. Split-time variability for finals and heats ranged between 0.9% and 2.5%
Cuenca-Fernández et al. [51]#	40 (40/0)		23.7 ± 3.8	Tier 4. Finalists, semi-finalists and the eight fastest non-qualifying swimmers from European short-course championships	The CV and relative change in performance were used to compare intra-individual performance progression between rounds and inter-individual differences between performance levels	Stroke rate, stroke length, swim velocity, pacing	Performance was maintained in 200 m compared to 100 m races, as well as in finalists compared to non-qualifying swimmers. In 100 m races, swimmers ↑ stroke rate, stroke length and clean-swimming velocity from heats/semi-finals to finals. In 200 m races, total time was the same between rounds
Šiljeg et al. [16]#	9 (0/9)		–	Tier 4. Female swimmers who competed at the 2004 and 2008 European Swimming Championships in the 100 m backstroke event	Situational success of female swimmers was measured through overall swim-time, start time (15 m), lap times per 25 m sections, swimming velocity, turn times (7.5 m), stroke frequency, stroke length and finish phase (5 m) were calculated	Stroke rate, stroke length, swim velocity,	Significant ↑ in overall time which was mostly accomplished with ↓ start and turn times. Progress in the anaerobic capabilities of four years older female swimmers is notable, indicated by ↓ variations in swimming velocity in the second half of the section
Gonjo et al. [62]#	10 (10/0)		17.4 ± 1.0	Tier 3. Specialized in backstroke	2 × 50 (1front crawl and 2 backstroke) at maximum swimming velocity	Stroke rate, stroke length, release phase, shoulder roll angle	Front crawl is faster than backstroke because of its higher stroke frequency due to the shorter absolute release phase
Fernandes et al. [46]	31 (15/16)		16.5 ± 0.5 15.9 ± 1.2	Tier 4. Elite with qualifying standards to participate in the World and European Junior Championships and regional and national standards	Video recorded in the sagittal plane when performing 25 m backstroke at maximal intensity	Stroke rate, stroke length, swim velocity, coordination	The elite swimmers’ performances were more unstable, but = intracycle velocity variation. Direct relationships were observed between mean velocity and stability, complexity with intracycle velocity variation. Backstroke performance is associated with velocity variability, with elite swimmers being able to control it through several adaptations
Craig et al. [47]#	20 (10/10)			Tier 4. Elite female swimmers during Olympic Swimming Trials of 1976 and 1984	Swimming performance (time) and stroke kinematic were measured in 100 and 200 m events	Stroke rate, stroke length, swim velocity	The ↑ velocity was accounted for by ↑distance per stroke. The finalists achieved ↑ distances per stroke than did the slower group

N = newtons, m = metres, m/sec = metres/second, min = minutes; CV = coefficient of variation. Swimmer classification: Tier 0 Sedentary, Tier 1 Recreationally Active, Tier 2 Trained/Developmental; Tier 3 Highly Trained/National Level; Tier 4 Elite/International Level; Tier 5 World Class according to the framework of McKay et al. [38]

#Articles including more than one stroke technique

### Characteristics of the Participants

The characteristics of the participants of the studies included in the systematic review are shown in Tables 1 and 2 (total sample size n = 507 participants, n = 188 women). A total of 14 studies [6, 11, 26–31, 46–48, 53, 55, 59] included both men and women, 4 studies included only women [16, 32, 52, 56] and the remainder included only male participants [12, 17, 18, 33–37, 41, 45, 48–51, 54, 58, 60]. All swimmers were classified as Tier 2 (Local-level representation), Tier 3 (Highly Trained/National Level) or Tier 4 (International Level) according to the classification framework of McKay et al. [38].

### Methodological Quality and Risk of Bias

Tables 3 and 4 summarize the quality and risk of bias of the studies included. The mean score was 11 ± 2 points (low risk of bias). One study had a score of 0–5 (high risk of bias) [47], 11 studies had a score between 6 to 10 points (satisfactory risk of bias) [12, 16, 28, 32, 34–37, 48, 53, 58], and 23 studies with 11–16 points (low risk of bias) [6, 11, 17, 18, 26, 27, 29–31, 33, 41, 46, 49, 50, 50–52, 54–57, 59, 62].

**Table 3:** Quality assessment of physiological and anthropometry articles on backstroke swimming

Study	Publication Year	1	2	3	4	5	6	7	8	9	10	11	12	13	14	15	16	Total
Carvalho et al. [26]	2020	*	*	*		*	*	*	*	*	*	*	*	*		*		13
Vescovi et al. [31]	2021	*	*	*		*	*	*	*	*	*	*	*	*				13
Ozkadi et al. [27]	2022	*	*	*		*	*	*	*	*	*	*	*	*		*		13
Sammoud et al. [30]	2019	*	*	*		*	*	*	*	*	*	*	*	*		*		13
Rejman et al. [29]	2003	*	*	*			*	*	*	*	*	*	*	*		*		12
Neiva et al. [33]	2011	*	*	*		*	*		*	*	*	*	*	*				11
Barbosa et al. [11]	2006	*	*	*			*	*	*	*	*	*	*	*				11
Capelli et al. [34]	1998	*	*	*				*	*	*	*	*	*	*				10
Barbosa et al. [12]	2008	*	*	*				*	*	*	*	*	*	*				10
Smith et al. [36]	1988	*	*	*			*	*	*	*	*	*	*					10
Klentrou & Montpetit [28]	1992	*	*	*			*		*	*	*	*	*					9
Makar & Bielec [32]	2013	*	*	*				*	*	*	*	*	*					9
Holmér [35]	1974							*	*	*	*	*	*	*				7
di Prampero [37]	2008		*							*	*	*	*	*				6

Checklist from Hindle et al. [23, 24]. The items were the follow: (1) study design was stated clearly; (2) the study objective/purpose is clearly stated; (3) the study has a clearly testable hypothesis; (4) the study clearly states the inclusion criteria for participants; (5) the characteristics of the population are well detailed; (6) the study population is representative of the intended population for which the research is aimed; (7) a justification for the selection of the sample/study population size was provided; (8) the methods used throughout testing are well detailed; (9) the measurement tools used throughout the study are reliable and have been validated; (10) detail on the statistical methods used was provided; (11) the statistical methods used to analyze the data were appropriate; (12) the results of the study are well detailed; (13) the information provided in the paper is sufficient to allow the reader to make an unbiased assessment of the study findings; (14) confounding factors within the study are identified; (15) study funding/conflicts of interest were acknowledged; and (16) limitations to the study were identified. Low risk of bias (11–16), Satisfactory risk of bias (6–10), High risk of bias (0–5)

**Table 4:** Quality assessment of kinematics and swimming velocity articles

Study	Publication Year	1	2	3	4	5	6	7	8	9	10	11	12	13	14	15	16	Total
Cuenca-Fernández et al. [51]	2023	*	*	*		*	*	*	*	*	*	*	*	*		*	*	14
Bartolomeu et al. [54]	2022	*	*	*	*	*	*	*	*	*	*	*	*	*		*		14
Gonjo et al. [41]	2018	*	*	*	*	*	*	*	*	*	*	*	*	*		*		14
Chollet et al. [49]	2008	*	*	*	*	*	*	*	*	*	*	*	*	*				13
Fernandes et al. [46]	2022	*	*	*		*	*	*	*	*	*	*	*	*		*		13
Gonjo et al. [60]	2022	*	*	*		*	*	*	*	*	*	*	*	*		*		13
Gonjo, et al. [45]	2021	*	*	*		*	*	*	*	*	*	*	*	*	*			13
Gonjo, et al. [50]	2020	*	*	*			*	*	*	*	*	*	*	*		*		12
Veiga et al. [57]	2016	*	*	*			*	*	*	*	*	*	*	*		*		12
Cortesi et al. [6]	2012	*	*	*		*	*	*	*	*	*	*	*	*				12
Gonjo et al. [62]	2020	*	*	*		*	*	*	*	*	*	*	*	*				12
Hellard et al. [56]	2008	*	*	*		*	*	*	*	*	*	*	*	*				12
Oliveira et al. [52]	2023	*	*	*			*	*	*	*	*	*	*	*		*		12
Morais et al. [17]	2022	*	*	*			*	*	*	*	*	*	*	*		*		12
Skorski [18]	2014	*	*	*	*		*	*	*	*	*	*	*	*				12
Lerda et al. [55]	2005	*		*		*	*	*	*	*	*	*	*	*				11
Lerda and Cardelli [58]	2003		*	*				*	*	*	*	*	*	*				9
Šiljeg et al. [16]	2011	*	*	*			*	*	*	*	*	*						9
Chollet et al. [48]	1996		*	*			*	*	*	*	*	*	*					9
Kolmogorov and Duplishcheva [53]	1992		*	*			*		*	*			*					6
Craig et al. [47]	1985									*	*	*	*	*				5

Checklist from Hindle et al. [23, 24]. The items were the follow: (1) study design was stated clearly; (2) the study objective/purpose is clearly stated; (3) the study has a clearly testable hypothesis; (4) the study clearly states the inclusion criteria for participants; (5) the characteristics of the population are well detailed; (6) the study population is representative of the intended population for which the research is aimed; (7) a justification for the selection of the sample/study population size was provided; (8) the methods used throughout testing are well detailed; (9) the measurement tools used throughout the study are reliable and have been validated; (10) detail on the statistical methods used was provided; (11) the statistical methods used to analyze the data were appropriate; (12) the results of the study are well detailed; (13) the information provided in the paper is sufficient to allow the reader to make an unbiased assessment of the study findings; (14) confounding factors within the study are identified; (15) study funding/conflicts of interest were acknowledged; (16) limitations to the study were identified. Low risk of bias (11–16), Satisfactory risk of bias (6–10), High risk of bias (0–5)

## Discussion

This systematic review provides reference values for blood lactate, kinematics, race pace, performance testing and anthropometric testing in 50, 100, and 200-m swimming. This information will be useful for developing coaching guidelines and backstroke-specific swimming testing protocols.

The normative data provided are limited to the included studies, which have inherent variability in study design, sample size, and participant characteristics. The methodological quality assessment of the included studies, based on the Hindle scale, yielded a mean score of 11 ± 2 points, categorizing most studies as having a low risk of bias. Among the 35 studies reviewed, 23 were classified as low risk (11–16 points), 11 had a satisfactory risk (6–10 points), and 1 study exhibited a high risk of bias (0–5 points).

### Physiological and Mechanical Parameters

Backstroke swimming performance is determined by a complex interplay of metabolic and mechanical factors. Efficient energy utilization and stroke mechanics are key in maintaining high speeds with minimal energy cost. The evidence suggests that maximizing stroke length while maintaining an effective SR can enhance performance, particularly in longer events like the 200-m backstroke. Understanding the physiological parameters is essential for unraveling the complexities behind variations in backstroke swimming performance. Physiological parameters, such as VO_2peak_, VO_2max_, lactate levels, and energy cost per stroke, provide valuable insights into the metabolic demands of backstroke swimming [28, 34]. Coaches can use these normative values to assess an athletes' aerobic capacity and design training regimens that address any disparities relative to these benchmarks. Fourteen studies (n = 14, 41%) assessed physiological parameters in this systematic review (Table 1). Blood lactate concentration was evaluated in several testing, training and competition settings. Logistic and accessibility constraints have limited the number of studies that have reported post-race blood lactate concentrations. For example, the available evidence shows blood lactate concentrations ranging from 9.1–11.1, 12.4–13.9, and 13.0–14.0 mmol L^−1^ for 50, 100, and 200-m/yard backstroke events, respectively [31, 34]. Vescovi et al. [31] suggest that estimated anaerobic contributions to maximal exercise are inversely related to exercise duration, and because of the short duration of 50 m races, the ATP-PC system is able to supply a substantial proportion of the energy needed for these events; thus blood lactate concentration was lower in comparison with 100–200 m events, not only in backstroke. Further evaluation of blood lactate concentrations across a range of training sets and competitive distances would be useful for backstroke swimmers of all performance levels.

Analysis of blood lactate in testing and training settings is more common. The anaerobic threshold during a 5 × 200 m test had values of 3.9 ± 1.1 mmol L^−1^ in 15 backstroke male and female international swimmers at a speed of 1.35 ± 0.09 m s^−1^ [5], showing good agreement with the velocity at 4 mmol L^−1^. Some researchers and coaches use fixed blood lactate values such as 4 mmol L^−1^ to assess the anaerobic threshold. However, a fixed blood lactate does not account for substantial interindividual differences and may frequently underestimate (in anaerobically-trained subjects) or overestimate (in aerobically-trained athletes) real endurance capacity. The maximal blood lactate concentration during an incremental test (i.e. 5 × 200 m) was between 5.1 and 12.6 mmol L^−1^, with a mean of ~ 11.0 mmol L^−1^ , in a female of the Polish National Junior Swimming Team over four consecutive seasons [32]. Monitoring physiological parameters such as anaerobic threshold or maximal blood lactate concentration during an incremental swimming test is useful for detecting changes in selected indicators of performance factors, but in world-ranked swimmers, these indicators are only modestly associated with competition performance [39].

The energetics of swimmers is most commonly measured in the pool by backward extrapolation or a snorkel device [11, 40]. Studies have reported VO_2peak_/VO_2max,_ values ranging between 52.2 ± 5.9 and 59.3 ± 5.2 ml kg^−1^ min^−1^ for males (N = 22) and females (N = 16), respectively, measured by a 400-m maximal swim test [28], and between 51.6 and 62.3 ml kg^−1^ min^−1^ in 20 elite male college swimmers measured by an incremental test [34]. These differences underscore the importance of considering sex-specific physiological characteristics when evaluating performance and designing training programs.

Energetics and efficiency are key components of swimming performance. In a classical study by Holmér [35], backstroke and freestyle strokes were the most economical styles. Subsequently, Capelli et al. [34] assessed the energy cost of swimming and concluded that energy cost was a continuous function of the speed in the four strokes, increasing exponentially in backstroke and crawl. These investigators reported energy cost values of 0.84 kJ m^−1^ and 1.47 kJ m^−1^ at 1 m s^−1^ and 1.5 m s^−1^, respectively in backstroke. Smith et al. [36], reported a linear increase in oxygen cost with speed, and this variable at 1.1 m s^−1^ was related to 400-m performance, while submaximal oxygen cost was also associated with 100-m and 200-m performance. Barbosa et al. [11] analyzed the relationships between energy cost, speed, and stroke mechanics in all four strokes in elite swimmers. Freestyle was the most economical among the competitive swimming strokes, followed by backstroke, butterfly, and breaststroke at all selected velocities (1.0, 1.2, 1.4, and 1.6 m s⁻^1^). Gongo et al. [41] also concluded that front crawl is less costly than backstroke, and limbs motion in front crawl is more effective than in backstroke. A lower energy cost in backstroke implies a reduction in energy expenditure at higher speeds, provided the swimmer employs good technique along with suitable VO_2max_ and lactate threshold levels. These requirements make energy cost a key factor for swimming success. These findings are consistent with Barbosa et al. [53], who stated that backstroke was the second most efficient competitive style after freestyle at all selected velocities. Coaches should focus on training drills and exercises that increase stroke length and decrease energy cost, especially in the 200-m backstroke, as affirmed by Barbosa et al. [12].

In backstroke, increasing SR is typically more challenging compared to front crawl. This is primarily due to the nature of arm coordination, where the propulsive phases do not overlap as they do in front crawl. In front crawl, swimmers can maintain a higher SR by coordinating arm movements to ensure continuous propulsion, minimizing the impact of resistance. In contrast, backstroke has a more distinct catch and recovery phase, which limits the degree to which SR can be increased without causing inefficiencies or excessive energy expenditure. However, stating that an increase in SR is the only option for improving performance in backstroke is somewhat reductive. While SL plays a crucial role in optimizing efficiency, elite swimmers may also enhance performance by refining stroke technique, optimizing body position, and improving muscular endurance to sustain a higher SR without excessive fatigue. Additionally, factors such as drag reduction and better kick efficiency can contribute to overall performance gains. Thus, while increasing SL is a key factor in backstroke efficiency, improvements in SR, technique, and biomechanics should not be overlooked.

Critical speed/power is moderately correlated (r = 0.39–0.80) with other metabolic parameters [42], so for coaches this parameter could be used for evaluation of the aerobic status [43]. Critical speed can be determined without access to dedicated sports science support, equipment and consumables. di Prampero et al. [37] reported that critical speed was 16% lower in backstroke compared to the vVO_2max_. Neiva et al. [33] detailed critical speeds in highly trained male swimmers, noting their values were lower (5.5%) than 100-m performance (1.53 ± 0.05 vs. 1.61 ± 0.06 m s^−1^) but highly correlated (r = 0.81; p = 0.015). It is incumbent on coaches and swimming scientists to have a clear strategy and plan for measuring critical speed with reference to other performance constructs including aerobic speed, lactate threshold, vVO_2_max, VO_2_max, and race pace profiles.

In summary, there is substantial information on blood lactate concentrations with backstroke swimming, but data on cardiorespiratory measures (e.g. VO_2_ max, swimming efficiency and energy costs) are in comparison more limited. Blood lactate testing can be conducted in competitions, but the other physiological measures are limited to the training and research environments. Coaches and swimming scientists can employ critical speed and related assessments to provide useful information on both energetics and the kinematic measures of SR and SL. Advancements in digital technology are enabling new insights into swimming training and racing using computer modelling [63], to complement more traditional pool-based assessments.

### Anthropometry

Anthropometric characteristics play a crucial role in backstroke performance, with factors such as height, arm span, and limb length influencing stroke mechanics and propulsion [13, 14]. Taller swimmers with longer limbs generally exhibit greater stroke lengths, which can contribute to improved efficiency and reduced drag. Research highlights that elite backstroke swimmers tend to have specific anthropometric advantages that align with stroke requirements, emphasizing the importance of talent identification and specialized training approaches. Another factor that influences the backstroke performance is the anthropometric characteristics of the swimmer. There is a long history of the importance of anthropometric and somatic features in swimming performance [29]. In addition, the kinematics of swimming are affected by the effect of anthropometric parameters. Ozkadi et al. [27] analyzed the anthropometric variables of 40 teenage girl swimmers with a mean age of 16.5 years old, concluding that body height, hand and foot lengths were dominant variables for the 50-m backstroke. Sammoud et al. [30] showed in teenagers that the strongest predictors of the 100-m event were the sitting height, leg length, and two girths (forearm and arm relaxed girth). For that reason, anthropometric variables are important factors for identifying and developing talent and influencing swimming performance [30, 44]. Results related to anthropometric variables are presented in Table 1. Technological advancements in body imaging and morphology including DXA [64] and 3-D scanning [65] provide the opportunity to undertake comprehensive profiling for the purposes of talent identification, nutritional interventions, strength training, swim-suit design, training evaluation, and technique modification. Future studies should further explore how these physical attributes interact with training adaptations to enhance competitive performance [27–30].

### Kinematics

It is well known that different swimming velocities involve various modifications in SR and SL—when the velocity is increased there are corresponding decreases in body roll, shoulder roll, and hip roll. However, these modifications are primarily related to changes in SR [45]. Fernandes et al. [46] examined the variability of backstroke velocity in swimmers of both elite and national levels with reference to sprint performance. Elite swimmers perform a higher SR (0.82 ± 0.07 vs. 0.71 ± 0.09 cycles s^−1^) and stroke index (2.89 ± 0.37 vs. 2.57 ± 0.41 m^2^ s^−1^ cycle) for similar SL values (1.88 ± 0.16 vs. 1.90 ± 0.17 m cycle^−1^). Both groups presented a backstroke catch-up coordination, but elite swimmers displayed a lower upper limb time lag (− 8.4 ± 5.0 vs. − 11.2 ± 6.0). Coaches and support staff can employ these kinematic measures for the purposes of evaluation and training prescription of backstroke swimmers. Both studies were carried out in a 25 m indoor pool.

**Fig. 2:**
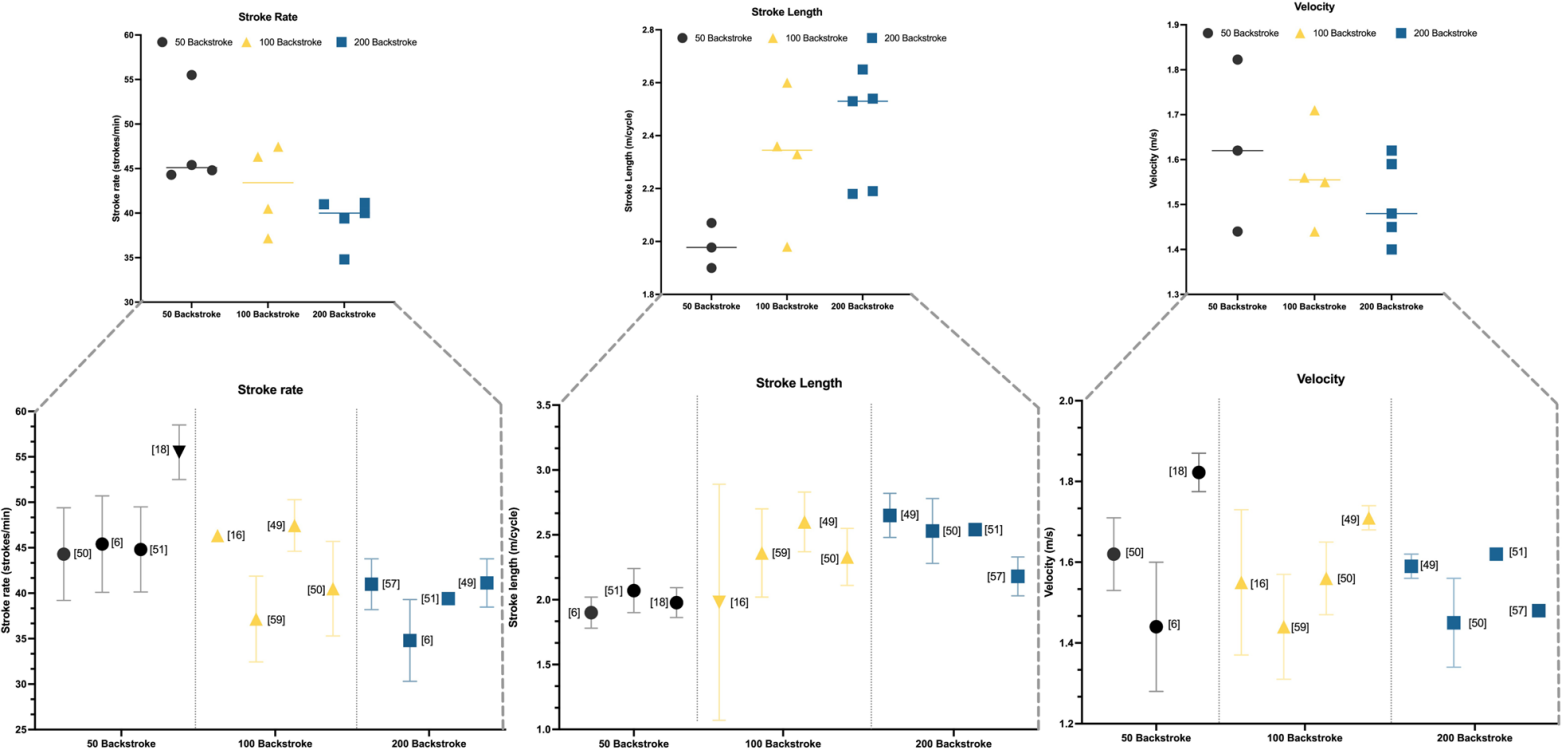
Relationship between stroke rate (SR), stroke length (SL) and velocity 50–100 and 200 m

Kinematic data have been collected in either 25 m or 50 m pools. The studies by Cortesi et al. [6], Gonjo et al. [43], Fernandes et al. [44], Chollet et al. [47], Gonjo et al. [48], Bartolomeu et al. [52], and Lerda et al. [52] were conducted in a 25 m pool, while the rest were conducted in a 50 m pool. Only the study by Smith et al. [36] was conducted in both 25 m and 50 m pools. Stroke kinematic characteristics including SR and SL vary by race distance and race duration [19]. Craig et al. [47] compared the U.S Olympic trials of 1976 and 1984. In backstroke, the faster speeds of the 100-m events relative to the 200-m distance were characterized by a higher SR and a shorter SL for both men and women. These results are in line with other studies by Chollet et al. [48], Chollet et al. [49] and Gonjo et al. [50] for males (100-m backstroke event: 37–47 cycles min^−1^ and speed 1.55–1.71 m s^−1^; 200-m backstroke event: 34–41 cycles min^−1^ and speed 1.40–1.62 m s^−1^). Barbosa et al. [12] similarly reported that increases in SR promoted increases in speed up to a given frequency. However, in contrast increases in the SL did not promote increases in the swimming speed of backstroke. In addition, speed increases were moderately associated with increases in SR and SL, even when controlling for the effect of SL (r = 0.64) and SR (r = 0.50), respectively. Results of kinematics parameters for 50 m, 100 m, and 200 m distances are shown in Fig. 2.

During international championships such as the European Short-course Championships [51], swimmers increased their SR by ~ 4% from heats/semi-finals to finals, and their SL and clean-swimming speed were also higher between rounds for backstroke events. It appears that improvements and superiority in stroke mechanics are reflected in the SR and SL used to swim a race. Fernandes et al. [46] compared elite and good-level swimmers and showed that elite swimmers presented higher SR (0.82 ± 0.07 versus 0.71 ± 0.09 cycles s^−1^) and stroke index (2.89 ± 0.37 versus 2.57 ± 0.41 m^2^ s^−1^ per cycle) for similar SL values (1.88 ± 0.16 versus 1.90 ± 0.17 m^2^ s^−1^ per cycle) compared to good-level swimmers. Both groups presented a backstroke catch-up coordination, but elite swimmers displayed lower upper limb time lag (− 8.4 ± 5.0% versus − 11.2 ± 6.0%). For elite swimmers, SR, intracycle velocity variation and the second down sweep phases were good mean speed predictors (*r* = 0.736). For good-level swimmers, the model was validated (*r* = 0.728), with SR and second up sweep being good mean velocity predictors.

In the 50-m sprint event, kinematics aspects such as SR and SL showed similar values in most studies in the 50-m backstroke event. The SR values in the studies analyzed [6, 47, 48] were ~ 45 cycles min^−1^ at speeds ranging from 1.44 to 1.62 m s^−1^. However, Morais et al. [17] and Oliveira et al. [52] showed higher SR values between 52 and 56 cycles min^−1^ but at higher maximal speeds (1.90–1.68 m s^−1^ and 1.82–1.60 m s^−1^ for faster and slower swimmers, respectively). The SL values ranged from 1.90 to 2.07 m cycle^−1^ [16, 48, 50]. In the study by Gonjo et al. [50], when swimmers swam at 93% of maximal 50-m speed, SR was reduced (37.5 ± 5.3 cycles min^−1^) while SL increased to 2.3 ± 0.2 m cycle^−1^. Lower top velocities were showed in the work of Kolmogorov and Duplishcheva [53], ranging from 1.20 to 1.35 m s^−1^ in female swimmers. In addition, the faster male swimmers did not differ in SR and SL compared to slower swimmers [17], but there was a higher SR for faster female swimmers (54–52 cycles min^−1^). It appears that sex-based differences are evident in the changes in kinematics and from good to elite swimmers. Bartolomeu et al. [54] showed that all strokes and variants presented contralateral limb asymmetries. Backstroke was the most asymmetric stroke, followed-up by butterfly stroke, front crawl, and breaststroke, although no substantial associations were identified between asymmetries and velocity in sprint swimming. In the 100-m backstroke event, the typical values of SR for male swimmers were between 37 and 47 cycles min^−1^ at speeds ranging between 1.55 and 1.71 m s^−1^, being higher when the speed of 100-m was faster [16, 36, 48, 49, 55]. In the case of female swimmers, the SR was 45.6 ± 3.4 cycles min^−1^ at 1.51 ± 0.03 m s^−1^ [48]. Regarding SL, the values ranged between 1.98 and 2.60 m cycle^−1^ for speeds between 1.55 and 1.71 m s^−1^ [16, 36, 48, 49]. For female swimmers, the values were 2.28 ± 0.18 m cycle^−1^ at 1.51 ± 0.03 m s^−1^ [48].

Finally, in the 200-m backstroke event, the studies showed similar SR, SL, and speed values. The SR values ranged between 34 and 41 cycles min^−1^ at speeds between 1.40 and 1.62 m s^−1^ [6, 48–50]. For female swimmers, the values ranged between 38.7 and 41.3 cycles min^−1^ at 1.41 to 1.49 m s^−1^ [48, 50, 56]. The SL values ranged between 2.10 and 2.65 m cycle^−1^ at speeds between 1.40 and 1.62 m s^−1^ [6, 48, 50]. In the case of only female swimmers, the values ranged between 2.18 and 2.30 m cycle^−1^ at speeds between 1.41 to 1.49 m s^−1^ [48, 50, 56]. In addition, female Olympic swimmers present higher speeds than national swimmers, 1.48 ± 0.01 and 1.41 ± 0.03 m s^−1^, respectively. Moreover, these swimmers present higher SR values, 41.0 ± 2.8 and 38.7 ± 2.9 cycles min^−1^. However, national swimmers present higher SL values, 2.18 ± 0.15 and 2.20 ± 0.15 m cycle^−1^, respectively. All these data correspond to the first 100 m of the 200 m event [56]. These data provide the opportunity for developing SR reference ranges by event (50-m, 100-m, 200-m), sex (male, female) and for use by coaches and support staff of backstroke swimmers.

Another kinematic factor related to swimming race performance is the starts and turns, particularly the relationships between the distances traveled underwater during the start and turn segments [57]. Overall, 200-m race times were largely related to longer starting distances in the women’s butterfly events, longer turn distances in men’s and women’s backstroke and women’s butterfly events, and shorter turn distances in women’s freestyle events. Changes in the start or turn velocities could elicit moderate improvements in time in most of the 100-m events, whereas modifications in the start or turn distances (especially in the last turn) could provide elite swimmers with time improvements of practical importance in the 200-m events. Faster backstroke swimmers were characterized by a greater kicking frequency coupled with specific segmental differences related to a lower knee range of motion in the upkick and downkick positions [59]. However, no sex differences were detected in the kicking velocity and kinematic variables when data were normalized for the swimmers’ height [59]. Stroke-specialists showed faster underwater speeds in front crawl and backstroke than individual medley swimmers [60]. Elite coaches should consider examining the start and turn distances travelled by swimmers, as extending (or shortening) the underwater swim could substantially increase their chances of improving performance times in backstroke in 100-m or 200-m.

### Pacing Strategies

Pacing strategies vary across different race distances in backstroke events, with swimmers adopting distinct approaches to optimize performance outcomes. In the 50-m event at major competitions swimmers adopt an all-out effort, with the first key moment point at the start (S0-15-m) and the second key moment at the finish (S45–50 m) [51]. Clearly, race pace is also affected when a competition is held in a short course swimming pool [51]. For pacing in the 100-m backstroke, it is common to observe a positive pacing profile, with the first lap faster than the second [3]. In the 200-m backstroke event, various pacing strategies are employed across different competitive levels. The most prevalent strategies include the parabolic approach, characterized by a fast start, an evenly paced mid-section, and a fast endspurt, as well as the fast-start even strategy, involving a fast beginning followed by a relatively consistent pace [3]. Skorski et al. [18] analyzed the reproducibility of pacing in elite swimmers during competitions and compared heats and finals in 35 elite swimmers. Swimmers adopted a fast-start even profile, with a substantially faster first section in all bouts, as well as a faster second split time compared with splits 3 and 4. There was a 7.6 ± 0.3% mean change between laps 1 and 2, 2.1 ± 0.3% between laps 2 and 3, and 0.2 ± 0.5% between laps 3 and 4 [51].

A diverse pattern of pacing strategies is employed by swimmers in the 200 m backstroke event across different competitive levels: a parabolic approach and the fast-start even strategy. The parabolic approach is characterized by a rapid start, followed by an evenly paced mid-section, and a strong finish. On the other hand, the fast-start even strategy involves a rapid initial burst of speed followed by a more consistent pace throughout the race [3, 61]. Understanding the interplay between SR, SL, and pacing dynamics informs race strategy and tactical decision-making for backstroke swimmers competing at elite levels.

### Practical Implications and Future Research

There are distinctive physiological, biomechanical patterns for all backstroke events from 50-m to 200-m. The data from the current research provide athletes and coaches with normative blood lactate, kinematics, race pace, and anthropometric reference values for comparison purposes, providing guidelines for swimmers to reach their best performance. Coaches and practitioners should also consider individual variations in anthropometric characteristics when selecting a backstroke swimmer (body height, hand and foot length in 50-m and sitting height, leg length, and forearm and arm girths in 100–200-m). In addition, these data provide a basis for designing training programs and technique refinement strategies for backstroke swimmers. While consistent patterns emerge across studies, variations in outcomes underscore the complexity of backstroke performance, and the need for further investigation to develop reference values, elucidate underlying mechanisms, and optimize training strategies. By synthesizing findings from multiple studies, we have provided a comprehensive overview of the current state of knowledge on backstroke performance and areas for future research and refinement.

### Study Limitations

This systematic review has some limitations. The largely observational nature of the studies restricts causal inferences between biomechanical, physiological, and anthropometric factors and backstroke performance. Additionally, inconsistencies in study design, sample size, and participant classification introduce potential selection bias.

The reliance on observational studies and retrospective analyses may introduce inherent biases and limit the generalizability of the findings to broader populations of backstroke swimmers. Furthermore, some data have been collected and published in scientific journals several years ago and probably do not fit with the new requirements of backstroke swimmers for this reason. Moreover, the specificity of the search strategy may have led to the omission of relevant studies using alternative terminology, despite efforts to refine the search and manually screen references. Future research should address the current limitations by incorporating prospective study designs, standardized methodologies and assessment protocols, and larger, more diverse cohorts of swimmers to enhance the validity, reliability, and overall robustness of findings in backstroke swimming performance. Moreover, interventional approaches should be explored, and additional factors such as psychological variables, environmental conditions, and technological innovations should be evaluated to provide a more comprehensive and holistic understanding of elite-level backstroke performance.

## Conclusion

This review provides valuable insights into the biomechanical and physiological determinants of elite backstroke swimming performance, shedding light on the multifactorial nature of competitive success in these events. Most studies were on highly-trained national-level swimmers rather than elite or world-class. Post-race blood lactate concentrations at different events are provided for backstroke (50-m was lower in comparison with 100–200 m events), as well as different SR reference ranges by event (50-m, 100-m, 200-m), anthropometric profiles (swimmers’ height and hand, foot, leg length), and pacing profiles (50-m: all-out trend; 100-m: positive profile; 200-m: parabolic approach or fast-track strategy). These reference values will inform development of specific resources to help coaches and support staff achieve the best performance of a backstroke swimmer.

## Data Availability

All data and material reported in this systematic review are from peer-reviewed publications.

## Abbreviations

ATP-PC: Adenosine triphosphate–phosphocreatine
PRISMA: Preferred Reporting Items for Systematic reviews and Meta-Analyses
VO_2max_: Maximum oxygen consumption in mlO_2_ kg^−1^ min^−1^
VO_2peak_: Peak oxygen consumption in mlO_2_ kg^−1^ min^−1^
vVO_2max_: Swimming velocity at maximum oxygen consumption
S0–15-m: Segment from 0 to 15 m in a swimming event
S45–50 m: Segment from 45 to 50 m in a swimming event
SR: Stroke rate
SL: Stroke length

## References

[1] Fina General Rules. Approved by the FINA Congress on 19 July 2019. Retrieved on https://resources.fina.org/fina/document/2021/01/12/35a6472c-8f19-416f-8c0c-0edef2773473/_logo_fina_general_rules_19.07.2019.pdf.

[2] Pyne DB, Sharp RL. Physical and energy requirements of competitive swimming events. Int J Sport Nutr Exerc Metab. 2014;24(4):351–9. 10.1123/ijsnem.2014-0047. 25029351 10.1123/ijsnem.2014-0047

[3] McGibbon KE, Pyne DB, Shephard ME, Thompson KG. Pacing in swimming: a systematic review. Sports Med. 2018;48(7):1621–33. 10.1007/s40279-018-0901-9. 29560605 10.1007/s40279-018-0901-9

[4] Menting SGP, Hendry DT, Schiphof-Godart L, Elferink-Gemser MT, Hettinga FJ. Optimal development of youth athletes toward elite athletic performance: how to coach their motivation, plan exercise training, and pace the race. Front Sports Active Living. 2019;1:14. 10.3389/fspor.2019.00014 PMC7739757 33344938

[5] Morais JE, Marinho DA, Arellano R, Barbosa TM. Start and turn performances of elite sprinters at the 2016 European Championships in swimming. Sports Biomech. 2019;18(1):100–14. 10.1080/14763141.2018.1435713. 29578384 10.1080/14763141.2018.1435713

[6] Cortesi M, Fantozzi S, Gatta G. Effects of distance specialization on the backstroke swimming kinematics. J Sports Sci Med. 2012;11(3):526–32. 24149363 PMC3737928

[7] Psycharakis SG, Sanders R. Body roll in swimming: a review. J Sports Sci. 2010;28:229–36. 10.1080/02640410903508847. 20131140 10.1080/02640410903508847

[8] Seiffert L, Boulesteix L, Carter M, Chollet D. The spatial–temporal and coordinative structures in elite male 100-m front crawl swimmers. Int J Sports Med. 2005;10:286–93. 10.1055/s-2004-821010 15795813

[9] Wakayoshi K, D’Acquisto LJ, Cappaert JM, Troup JP. Relationship between oxygen uptake, stroke rate and swimming velocity in competitive swimming. Int J Sports Med. 1995;16(1):19–23. 10.1055/s-2007-972957. 7713625 10.1055/s-2007-972957

[10] Termin B, Pendergast DR. Training using the stroke frequency—velocity relationship to combine biomechanical & metabolic paradigms. J Swim Res. 2000;14:9–17.

[11] Barbosa TM, Fernandes R, Keskinen KL, Colaço P, Cardoso C, Silva J, Vilas-Boas JP. Evaluation of the energy expenditure in competitive swimming strokes. Int J Sports Med. 2006;27(11):894–9. 10.1055/s-2006-923776. 16612740 10.1055/s-2006-923776

[12] Barbosa TM, Fernandes RJ, Keskinen KL, Vilas-Boas JP. The influence of stroke mechanics into energy cost of elite swimmers. Eur J Appl Physiol. 2008;103(2):139–49. 10.1007/s00421-008-0676-z. 18214521 10.1007/s00421-008-0676-z

[13] Alves M, Carvalho DD, Fernandes RJ, Vilas-Boas JP. How anthropometrics of young and adolescent swimmers influence stroking parameters and performance? A systematic review. Int J Environ Res Public Health. 2022;19(5):2543. 10.3390/ijerph19052543. 35270236 10.3390/ijerph19052543 PMC8909379

[14] Carvalho DD, Monteiro AS, Fonseca P, Silva AJ, Vilas-Boas JP, Pyne DB, Fernandes RJ. Swimming sprint performance depends on upper/lower limbs strength and swimmers’ level. J Sports Sci. 2023;41(8):747–57. 10.1080/02640414.2023.2239610. 37488696 10.1080/02640414.2023.2239610

[15] Marinho DA, Barbosa TM, Neiva HP, Silva AJ, Morais JE. Comparison of the start, turn and finish performance of elite swimmers in 100 m and 200 m races. J Sports Sci Med. 2020;19(2):397–407. 32390734 PMC7196746

[16] Šiljeg K, Leko G, Mikulić P. Situational success in 100-m backstroke event at the 2004 and 2008 European swimming championships. Sport Sci. 2011;4(2):28–31.

[17] Morais JE, Barbosa TM, Lopes T, Simbaña-Escobar D, Marinho DA. Race analysis of the men’s 50 m events at the 2021 LEN European Championships. Sports Biomech. 2022. 10.1080/14763141.2022.2125430. 36164890 10.1080/14763141.2022.2125430

[18] Skorski S, Faude O, Caviezel S, Meyer T. Reproducibility of pacing profiles in elite swimmers. Int J Sports Physiol Perform. 2014;9(2):217–25. 10.1123/ijspp.2012-0258. 23689199 10.1123/ijspp.2012-0258

[19] Craig AB Jr, Pendergast DR. Relationships of stroke rate, distance per stroke, and velocity in competitive swimming. Med Sci Sports. 1979;11(3):278–83. 522640

[20] González-Ravé JM, Hermosilla F, González-Mohíno F, Casado A, Pyne DB. Training intensity distribution, training volume, and periodization models in elite swimmers: a systematic review. Int J Sports Physiol Perform. 2021;16(7):913–26. 10.1123/ijspp.2020-0906. 33952709 10.1123/ijspp.2020-0906

[21] Nicol E, Pearson S, Saxby D, Minahan C, Tor E. Stroke kinematics, temporal patterns, neuromuscular activity, pacing and kinetics in elite breaststroke swimming: a systematic review. Sports Med Open. 2022;8(1):75. 10.1186/s40798-022-00467-2. 35674850 10.1186/s40798-022-00467-2 PMC9177912

[22] Moher D, Liberati A, Tetzlaff J, Altman DG, Group P. Preferred reporting items for systematic reviews and meta-analyses: the PRISMA statement. PLoS Med. 2009;6(7):e1000097. 10.1136/bmj.b2535. 19621072 10.1371/journal.pmed.1000097 PMC2707599

[23] Hindle BR, Lorimer A, Winwood P, Keogh JW. The biomechanics and applications of strongman exercises: a systematic review. Sports Med Open. 2019. 10.1186/s40798-019-0222-z. 31820223 10.1186/s40798-019-0222-z PMC6901656

[24] Hindle BR, Lorimer A, Winwood P, Keogh JW. A systematic review of the biomechanical research methods used in strongman studies. Sports Biomech. 2020;19(1):90–119. 10.1080/14763141.2019.1598480. 31132028 10.1080/14763141.2019.1598480

[25] Natera AO, Cardinale M, Keogh JW. The effect of high-volume power training on repeated high-intensity performance and the assessment of repeat power ability: a systematic review. Sports Med. 2020. 10.1007/s40279-020-01273-0). 32096112 10.1007/s40279-020-01273-0

[26] Carvalho DD, Soares S, Zacca R, Sousa J, Marinho DA, Silva AJ, Vilas-Boas JP, Fernandes RJ. Anaerobic threshold biophysical characterisation of the four swimming techniques. Int J Sports Med. 2020;41(5):318–27. 10.1055/a-0975-9532. 31975360 10.1055/a-0975-9532

[27] Özkadı T, Demirkan E, Can S, Alagöz I, Demir E. Contribution of motoric and anthropometric components to the fifty-meter four swimming styles: model approaches. Sci Sports. 2022;37(4):316.e1-316.e10.

[28] Klentrou PP, Montpetit RR. Energetics of backstroke swimming in males and females. Med Sci Sports Exerc. 1992;24(3):371–5. 1549032

[29] Rejman M, Nevill AM, Garrido ND, Rudnik D, Morais JE. Identification of key somatic features that are common and the ones that differ between swim strokes through allometric modeling. Front Sports Act Living. 2023;5:1308033. 10.3389/fspor.2023.1308033. 38107674 10.3389/fspor.2023.1308033 PMC10722254

[30] Sammoud S, Nevill AM, Negra Y, Bouguezzi R, Helmi C, Hachana Y. Key somatic variables in young backstroke swimmers. J Sports Sci. 2019;37(10):1162–7. 10.1080/02640414.2018.1546547. 30430909 10.1080/02640414.2018.1546547

[31] Vescovi JD, Falenchuk O, Wells GD. Blood lactate concentration and clearance in elite swimmers during competition. Int J Sports Physiol Perform. 2011;6(1):106–17. 10.1123/ijspp.6.1.106. 21487154 10.1123/ijspp.6.1.106

[32] Makar P, Bielec G. Lactate and glucose concentrations in assessing anaerobic capacity in an elite junior swimmer—a case study. Hum Mov. 2013;14(4):360–5. 10.2478/humo-2013-0044.

[33] Neiva HP, Fernandes RJ, Vilas-Boas JP. Anaerobic critical velocity in four swimming techniques. Int J Sports Med. 2011;32(3):195–8. 10.1055/s-0030-1268474. 21165797 10.1055/s-0030-1268474

[34] Capelli C, Pendergast DR, Termin B. Energetics of swimming at maximal speeds in humans. Eur J Appl Physiol Occup Physiol. 1998;78(5):385–93. 10.1007/s004210050435. 9809837 10.1007/s004210050435

[35] Holmér I. Energy cost of arm stroke, leg kick, and the whole stroke in competitive swimming styles. Eur J Appl Physiol Occup Physiol. 1974;33(2):105–18. 10.1007/BF00449512. 4430307 10.1007/BF00449512

[36] Smith HK, Montpetit RR, Perrault H. The aerobic demand of backstroke swimming, and its relation to body size, stroke technique, and performance. Eur J Appl Physiol. 1988;58:182–8. 10.1007/BF00636624. 10.1007/BF00636624 3203665

[37] di Prampero PE, Dekerle J, Capelli C, Zamparo P. The critical velocity in swimming. Eur J Appl Physiol. 2008;102(2):165–71. 10.1007/s00421-007-0569-6. 17901978 10.1007/s00421-007-0569-6

[38] McKay AK, Stellingwerff T, Smith ES, Martin DT, Mujika I, Goosey-Tolfrey VL, Sheppard J, Burke LM. Defining training and performance caliber: a participant classification framework. Int J Sports Physiol Perform. 2022;17(2):317–31. 34965513 10.1123/ijspp.2021-0451

[39] Pyne DB, Lee H, Swanwick KM. Monitoring the lactate threshold in world-ranked swimmers. Med Sci Sports Exerc. 2001;33(2):291–7. 11224820 10.1097/00005768-200102000-00019

[40] Hausswirth C, Bigard A, Le Chevalier J. The Cosmed K4 telemetry system as an accurate device for oxygen uptake measurements during exercise. Int J Sports Med. 1997;18:449–53. 9351691 10.1055/s-2007-972662

[41] Gonjo T, McCabe C, Sousa A, Ribeiro J, Fernandes RJ, Vilas-Boas JP, Sanders R. Differences in kinematics and energy cost between front crawl and backstroke below the anaerobic threshold. Eur J Appl Physiol. 2018;118:1107–18. 29556773 10.1007/s00421-018-3841-z

[42] Galán-Rioja MÁ, Gonzalez-Mohino F, Poole DC, González-Ravé JM. Relative proximity of critical power and metabolic/ventilatory thresholds: systematic review and meta-analysis. Sports Med. 2020;50:1771–83. 32613479 10.1007/s40279-020-01314-8

[43] Dekerle J, Sidney M, Hespel JM, Pelayo P. Validity and reliability of critical speed, critical stroke rate, and anaerobic capacity in relation to front crawl swimming performances. Int J Sports Med. 2002;23(02):93–8. 11842355 10.1055/s-2002-20125

[44] Morais JE, Silva AJ, Marinho DA, Lopes VP, Barbosa TM. Determinant factors of long-term performance development in young swimmers. Int J Sports Physiol Perform. 2017;12(2):198–205. 10.1123/ijspp.2015-0420. 27196954 10.1123/ijspp.2015-0420

[45] Gonjo T, Fernandes RJ, Vilas-Boas JP, Sanders R. Body roll amplitude and timing in backstroke swimming and their differences from front crawl at the same swimming intensities. Sci Rep. 2021;11(1):824. 10.1038/s41598-020-80711-5. 33436944 10.1038/s41598-020-80711-5 PMC7804020

[46] Fernandes A, Goethel M, Marinho DA, Mezêncio B, Vilas-Boas JP, Fernandes RJ. Velocity variability and performance in backstroke in elite and good-level swimmers. Int J Environ Res Public Health. 2022;19(11):6744. 10.3390/ijerph19116744. 35682325 10.3390/ijerph19116744 PMC9180488

[47] Craig AB Jr, Skehan PL, Pawelczyk JA, Boomer WL. Velocity, stroke rate, and distance per stroke during elite swimming competition. Med Sci Sports Exerc. 1985;17(6):625–34. 10.1249/00005768-198512000-0000. 4079732 10.1249/00005768-198512000-00001

[48] Chollet D, Pelayo P, Tourny C, Sidney M. Comparative analysis of 100 m and 200 m events in the four strokes in top level swimmers. J Hum Mov Stud. 1996;31:25–38.

[49] Chollet D, Seifert LM, Carter M. Arm coordination in elite backstroke swimmers. J Sports Sci. 2008;26(7):675–82. 10.1080/02640410701787791. 18409098 10.1080/02640410701787791

[50] Gonjo T, Narita K, McCabe C, Fernandes RJ, Vilas-Boas JP, Takagi H, Sanders R. Front crawl is more efficient and has smaller active drag than backstroke swimming: kinematic and kinetic comparison between the two techniques at the same swimming speeds. Front Bioeng Biotechnol. 2020;8: 570657. 33072727 10.3389/fbioe.2020.570657 PMC7543982

[51] Cuenca-Fernández F, Ruiz-Navarro JJ, Polach M, Arellano R, Born D-P. Short-course performance variation across all race sections: how 100 and 200 m elite male swimmers progress between rounds. Front Sports Act Living. 2023;5:1146711. 10.3389/fspor.2023.1146711. 37057072 10.3389/fspor.2023.1146711 PMC10086268

[52] Oliveira JP, Marinho DA, Barbosa TM, Sampaio T, Morais JE. Profile of female swimmers competing in the 50 m events at the 2021 LEN European Championships. Int J Perform Anal Sport. 2023;23(2):97–110.

[53] Kolmogorov SV, Duplishcheva OA. Active drag, useful mechanical power output and hydrodynamic force coefficient in different swimming strokes at maximal velocity. J Biomech. 1992;25(3):311–8. 10.1016/0021-9290(92)90028-y. 1564064 10.1016/0021-9290(92)90028-y

[54] Bartolomeu RF, Rodrigues P, Santos CC, Costa MJ, Barbosa TM. Is there any effect of symmetry on velocity of the four swimming strokes? Symmetry. 2022;14:12. 10.3390/sym14010012.

[55] Lerda R, Cardelli C, Coudereau JP. Backstroke organization in physical education students as a function of skill and sex. Percept Mot Skills. 2005;100(3 Pt 1):779–90. 10.2466/pms.100.3.779-790. 16060443 10.2466/pms.100.3.779-790

[56] Hellard P, Dekerle J, Avalos M, Caudal N, Knopp M, Hausswirth C. Kinematic measures and stroke rate variability in elite female 200-m swimmers in the four swimming techniques: Athens 2004 Olympic semi-finalists and French National 2004 Championship semi-finalists. J Sports Sci. 2008;26(1):35–46. 17896287 10.1080/02640410701332515

[57] Veiga S, Roig A, Gómez-Ruano MA. Do faster swimmers spend longer underwater than slower swimmers at World Championships? Eur J Sport Sci. 2016;16(8):919–26. 10.1080/17461391.2016.1153727. 26930126 10.1080/17461391.2016.1153727

[58] Lerda R, Cardelli C. Analysis of stroke organization in the backstroke as a function of skill. Res Q Exerc Sport. 2003;74(2):215–9. 10.1080/02701367.2003.10609083. 12848234 10.1080/02701367.2003.10609083

[59] Veiga S, Qiu X, Trinidad A, Dolek BE, de la Rubia A, Navarro E. Effect of the skill, gender, and kick order on the kinematic characteristics of underwater undulatory swimming in the dorsal position. J Hum Kinet. 2023;11(90):45–56. 10.5114/jhk/168600. 10.5114/jhk/168600 PMC10875688 38380311

[60] Gonjo T, Polach M, Olstad BH, Romann M, Born DP. Differences in race characteristics between world-class individual-medley and stroke-specialist swimmers. Int J Environ Res Public Health. 2022;19(20):13578. 10.3390/ijerph192013578. 36294159 10.3390/ijerph192013578 PMC9603436

[61] Robertson EY, Pyne DB, Hopkins WG, Anson JM. Analysis of lap times in international swimming competitions. J Sports Sci. 2009;27(4):387–95. 19214862 10.1080/02640410802641400

[62] Gonjo T, Fernandes RJ, Vilas-Boas JP, Sanders R. Upper body kinematic differences between maximum front crawl and backstroke swimming. J Biomech. 2020;98:109452. 10.1016/j.jbiomech.2019.109452. 31708239 10.1016/j.jbiomech.2019.109452

[63] Carvalho DD, Goethel MF, Silva AJ, Vilas-Boas JP, Pyne DB, Fernandes RJ. Swimming performance interpreted through explainable artificial intelligence (XAI)—practical tests and training variables modelling. Appl Sci. 2024;14(12):5218. 10.3390/app14125218.

[64] Almeida-Neto PF, Baxter-Jones A, de Medeiros JA, Dantas PMS, Cabral BGAT. Are there differences in anaerobic relative muscle power between upper and lower limbs in adolescent swimmers: a blinded study. Sports Med Health Sci. 2023;5(4):290–8. 10.1016/j.smhs.2023.09.005. 38314042 10.1016/j.smhs.2023.09.005 PMC10831378

[65] Oberhofer K, Knopfli C, Achermann B, Lorenzetti SR. Feasibility of using laser imaging detection and ranging technology for contactless 3D body scanning and anthropometric assessment of athletes. Sports (Basel). 2024;12(4):92. 10.3390/sports12040092. 38668560 10.3390/sports12040092 PMC11054930

